# An immunohistochemical study of canine spontaneous gastric polyps

**DOI:** 10.1186/s13000-014-0166-z

**Published:** 2014-09-18

**Authors:** Irina Amorim, Marian A Taulescu, Andreia Ferreira, Alexandra Rêma, Celso A Reis, Augusto M Faustino, Cornel Cătoi, Fátima Gärtner

**Affiliations:** Institute of Biomedical Sciences Abel Salazar (ICBAS), University of Porto, Rua Jorge Viterbo Ferreira nr.228, 4050-313 Porto, Portugal; Institute of Molecular Pathology and Immunology of the University of Porto (IPATIMUP), Rua Dr. Roberto Frias s/n, 4200-465 Porto, Portugal; Faculty of Veterinary Medicine, University of Agricultural Sciences and Veterinary Medicine, Cluj-Napoca, Romania; Faculty of Sciences of the University of Porto (FCUP), Rua do Campo Alegre 1021/1055, 4169-007 Porto, Portugal; Faculty of Medicine, University of Porto, Alameda Prof. Hernâni Monteiro, 4200- 319 Porto, Portugal

**Keywords:** Canine gastric polyps, COX-2, CDX2, *Helicobacter* spp, Ki67, p53

## Abstract

**Background:**

Gastric polyps (GP) are characterised by luminal overgrowths projecting above the plane of the mucosal surface that can be classified as non-neoplastic and neoplastic lesions. In humans, recent studies have drawn attention to the malignant potential of some of these lesions. However, gastric polyps are uncommon lesions in dogs.

**Findings:**

In this study, the presence of *Helicobacter* spp., the cellular proliferative activity, potential phenotypic alterations, COX-2 and p53 expression in canine spontaneous gastric polyps were investigated. The expression of these molecules was also studied in normal canine gastric mucosa in order to gain further knowledge of the significance of their loss or overexpression in gastric lesions.

**Conclusions:**

The normal expression of almost all the factors evaluated, along with the reduced proliferative activity is strongly suggestive that, in dogs, spontaneous gastric polyps are not only a rare finding but also of benign nature.

**Virtual Slides:**

The virtual slide(s) for this article can be found here: http://www.diagnosticpathology.diagnomx.eu/vs/13000_2014_166

## Introduction

Gastric polyps (GPs) are considered a heterogeneous entity broadly defined as luminal lesions projecting above the plane of the mucosal surface that can be biologically classified as non-neoplastic and neoplastic lesions. In humans, further classification relies on the evaluation of the histogenesis and neoplastic potential of these lesions [1]. Accordingly, among the non-neoplastic epithelial gastric polyps, three subtypes are recognized: fundic gland polyps; hyperplastic polyps (and variants) and neuroendocrine tumours (NET).

Fundic gland polyps represent the most common type and they are histologically characterized by cistically dilated fundic-type glands lined by flattened parietal cells with a degree of architectural distortion. The surface comprises foveolar type epithelium, which may appear atrophic [1]. The hyperplastic polyps are typically observed in the antrum and histologically consist in hyperplastic, elongated and branching foveolae set in an abundant oedematous and inflamed stroma. Cystic dilation of the pits is almost invariable present in the deeper portions and surface is lined by a single-layer of foveolar-type epithelium [1]. Hyperplastic polyps occur against a background of gastritis and its development is a consequence of an exaggerated mucosal response to injury. They are related to *Helicobacter pylori* (*H. pylori*) infection and some authors concluded that *H. pylori* eradication cause complete regression or significant decrease in its size. It is important to be aware that foci of dysplasia or intramucosal carcinoma can be encountered in these lesions [1,2]. Lastly, NET or the so called “carcinoid” tumours are histologically composed of nests, cords, tubules, and clusters of cells that predominantly interposed between the foveolar basis, without disturbing the overall polyp architecture [3].

Contrary to the documented progression in human colorectal cancer, the association of GPs with the development of gastric cancer is still not fully established but studies emphasize the malignant potential of these lesions. GPs are uncommon in dogs and are occasional incidental findings during endoscopy or postmortem examinations [4]. Sporadic canine GPs are rare and the relevant literature is scarce. Only recently Taulescu et al. [5] described the histopathological features of 15 lesions. Regarding non-neoplastic polypoid growths, the World Health Organization (WHO) for the classification of tumors in domestic animals presents a simplest scheme where only two entities are recognized: the hyperplastic (regenerative) and the inflammatory (benign lymphoid) polyps. The former are histologically identical to the human homonymous lesion and the gold standard of the later is the presence of a normal epithelium covering a granulation tissue core infiltrated by a variety of inflammatory cells, sometimes accompanied by lymphoid aggregates with prominent germinal centers [4].

Several studies trying to involve some markers for malignant transformation in human gastric hyperplastic polyps were performed however, a specific molecule that could identify which polyp will undergo malignant transformation has not yet been found. In the dog, not much is known with this regard.

Cyclooxygenase (COX) is the enzyme that allows synthesis of prostaglandins and other eicosanoids from arachidonic acid. COX-2 isoform plays an important role in gastric cancer development through apoptosis inhibition and increased cell proliferation. Although few studies reported the increased expression of COX-2 in canine cancers no data is available regarding COX-2 expression in canine gastric tissues [6].

Cellular kinetics is thought to participate in the progression towards gastric cancer. Ki-67 antibody is used to measure the proliferation index of a particular tissue assuming that higher Ki-67 reveals tumour cell activity and predicts the further behaviour of a specific pathology [7].

Malignant transformation of human hyperplastic GPs is associated with dysplasia, with p53 playing a crucial role in the process [8]. Overexpression of mutated p53 has been correlated with pathological parameters of tumour aggressiveness and poor prognosis in human gastric carcinoma [9].

According to Correa’s model [10], human gastric cancer develops in a multistep process from chronic active gastritis to intestinal metaplasia (IM), dysplasia, and finally, gastric cancer. IM is regarded as precursor lesion, consisting of the transdifferentiation of gastric mucosa to an intestinal phenotype, characterised by the aberrant expression of the homeobox transcription factor CDX2 [11]. Expression of CDX2 in canine GPs has never been characterised.

As uncommon lesions, the molecular properties and neoplastic potential of canine GPs remains an area of interest. The purpose of this study is to describe some molecular features of these lesions in order to better understand their biological behaviour. Additionally, the expression of these molecules in normal gastric mucosa (NGM) was investigated to evaluate the significance of their loss or overexpression in gastric lesions.

## Animals & methods

**Figure 1:**
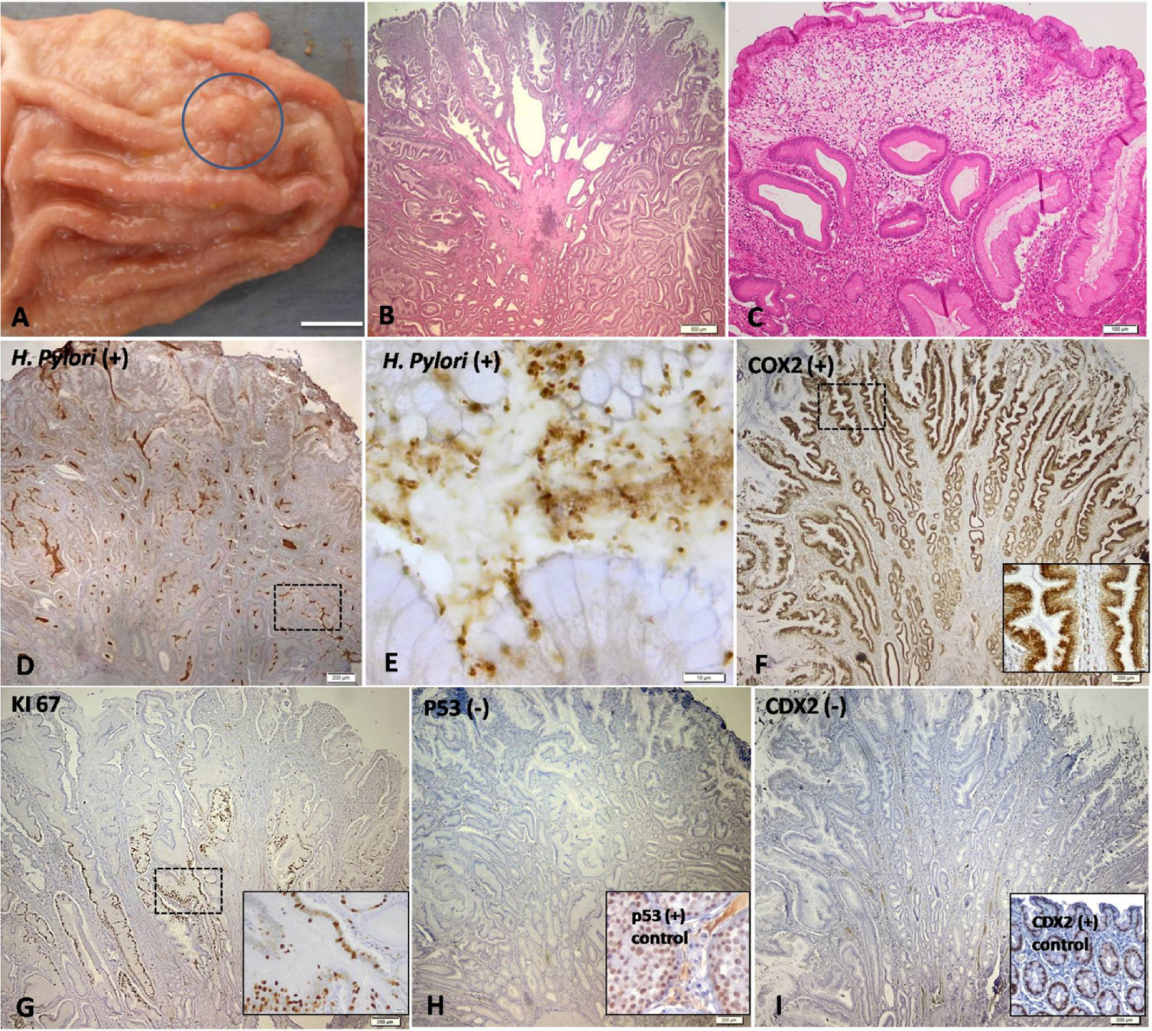
**Morphological and immunohistochemical features of the canine gastric polyps. (A)** Gross aspect; **(B)** Hyperplastic polyp; **(C)** Inflammatory polyp; **(D) (E)** *H. pylori* immunoexpression. **(F)** COX-2 immunoexpression; **(G)** Ki-67 immunoexpression; **(H)** p53 immunoexpression. Inset: intratubular seminoma (+control) **(I)** CDX2 immunoexpression. Inset: normal intestine (+control). Morphological and immunohistochemical features of the canine gastric polyps. **(A)** Gross aspect of the pyloric gastric mucosa showing a solitary polyp (delimited area) (case 9); **(B)** and **(C)** Microscopic examination of haematoxylin and eosin-stained slides revealing a hyperplastic polyp (case 4) and an inflammatory polyp (case 8). Bar = 500 μm and 100 μm, respectively. **(D)** Photomicrograph demonstrating the positive *H. pylori* expression in a hyperplastic polyp (case 2). Bar = 200 μm. **(E)** Detail of the previous case highlighting the large amount of *Helicobacter* spp. organisms inside the gastric glands. Bar = 10 μm. **(F)** Photomicrograph revealing strong and diffuse COX-2 expression into branched foveolar epithelium of the hyperplastic polyp (case 1). Bar = 200 μm. Inset presenting a detail of the COX-2 expression into foveolar epithelium; **(G)** Immunostaining of Ki-67 in the hyperplastic epithelium (case 3: 32.1%). Bar = 200 μm. Inset showing the detail of KI-67 expression in the isthmus and in the glands base. **(H)** Photomicrograph revealing a negative immunohistochemical reactivity of p53 in the hyperplastic polyp (case 2). Bar = 200 μm. Tissue from canine intratubular seminoma has been used as positive control for p53 (inset). **(I)** Negative CDX2 expression in the hyperplastic polyp (case 1). Bar = 200 μm. Tissue from canine normal intestine has been used as positive control for CDX2 (inset). Immunoperoxidase-diaminobenzidine stain with Mayer’s haematoxylin counterstain **(D-I)**.

Four normal canine antral gastric mucosa specimens and nine canine antral GPs (Figure 1A), received between 2007 and 2013, were selected from the archives of the Laboratory of Veterinary Pathology, ICBAS-UP (Portugal) and Pathology Department of FVM, Cluj-Napoca (Romania). Tissues were fixed in 10% buffered formalin and paraffin-embedded. Serial consecutive sections 3 μm-thick were made, one for routine histological diagnosis and the others for immunohistochemical studies.

Sections were independently examined by three pathologists. Normal tissues were considered as such according to Prachasilpchain et al. [10] and were negative for the presence of *Helicobacter* spp. (confirmed by modified Giemsa stain and anti-*H. pylori* immunohistochemistry). GPs were classified according to WHO classification for domestic animals diagnostic criteria [3].

For the immunohistochemical study, a panel of antibodies specific for several antigens was applied (Table 1). Novolink Max-Polymer detection system (Novocastra) was used according to the manufacturer’s instructions.

**Table 1:** **Antibodies used for canine gastric tissues immunohistochemistry**

**Marker**	**Clone**	**Supplier**	**Dilution**	**Antigen unmasking**	**Incubation period**	**Positive control**
*H. pylori*	Polyclonal	Zytomed, Deutschland	1:200	CB/PC	ON	Human gastric mucosa with *H. pylori*
COX-2	SP21	NeoMarkers, USA	1:75	CB/PC	ON	Canine mammary tumour
p53	Anti-p53	Novocastra, UK	1:400	CB/PC	ON	Canine intratubular seminoma
CDX2	AMT28	Novocastra, UK	1:300	CB/PC	ON	Canine normal intestine
KI-67	MIB-1	Dako, Denmark	1:50	CB/PC	ON	Canine high grade lymphoma

*CB* citrate buffer; PC pressure cooker.

*ON* overnight.

Detection of *Helicobacter* spp. was rated as: negative, no organisms, or positive, presence of *Helicobacter* spp. Bacterial density colonization was quantified: *+*, few (<10/400×); *++*, moderate (10-50/400×) and +++, large number of organisms (>50/400×). Bacteria location was recorded: 0 = absent; 1 = mucosal surface and gastric pits; 2 = 1 + lumen of gastric glands (400×) [12]. COX-2 immunoexpression was scored for the percentage of labelled cells (<25%; 25-50%; 51-75%; >75%) and labelling intensity (0, negative; +, weak; ++, moderate; +++, strong). The Ki-67 labelling index was defined as percentage of positive nuclei determined by counting at least 1000 nuclei in the selected fields (×400) [8]. Additionally, immunoreactivity was assessed considering the gastric glands divided in three zones: zone 1 (gastric pit); zone 2 (isthmus/proliferative zone) and zone 3 (gland base) [13]. p53 and CDX2 immunoreactivity were defined as positive when distinct nuclear staining was recognised in at least 10% of the cells [8,14].

## Findings

The available clinical data, histological classification of the lesions and main immunohistochemical results, considering the criteria proposed for each antibody, are summarised in Table 2.

**Table 2:** **Available data of the animals, histological classification of the canine gastric tissues and main immunohistochemical findings**

**Case**	**Breed**	**Gender/Age (years)**	**Histological diagnosis**	***Helicobacter*** **spp.**	**COX-2**		**KI-67 (%)**	**p53**	**CDX2**
					**SE**	**DG**			
1	Rottweiler	M/12	Hyperplastic polyp	+++^2^	>75%/+++	>75%/ +++	36,1^2,3^	-	-
2	Irish Setter	F/12	Hyperplastic polyp	+++^2^	>75%/+++	>75%/+++	31,4^2,3^	-	-
3	Poodle	F/12	Hyperplastic polyp	+++^2^	>75%/ +++	>75%/+++	32,1^2,3^	-	-
4	Argentine Mastiff	F/9	Hyperplastic polyp	++^2^	>75%/+++	>75%/ +++	28,9^2,3^	-	-
5	Rottweiler	F/14	Hyperplastic polyp	+^2^	>75%/+++	>75%/+++	30,9^2,3^	-	-
6	Mixed breed	F/16	Hyperplastic polyp	++^2^	>75%/+++	>75%/+++	16,7^2,3^	-	-
7	Poodle	M/13	Inflammatory polyp	++^2^	>75%/+++	>75%/+++	31,5^2,3^	-	-
8	German Shepherd	M/9	Inflammatory polyp	++^2^	>75%/+++	>75%/+++	10,8^2^	-	-
9	Boxer	M/10	Hyperplastic polyp	+^1^	>75%/+++	>75%/+++	4,6^2^	-	-
10	Mixed breed	M/2	NGM	0	>75%/+++	>75%/+	18,3^2^	-	-
11	Poodle cross	F/15	NGM	0	>75%/+++	>75%/++	23,6^2^	-	-
12	Boxer	M/4	NGM	0	>75%/+++	>75%/+	15,6^2^	-	-
13	Mixed breed	M/8	NGM	0	>75%/+++	>75%/++	22,8^2^	-	-

*M* male, *F* female.

COX-2: SE, surface epithelium; DG, deeper glands. >75% of immunopositive cells, + weak, ++ moderate, +++ strong intensity.

*Helicobacter* spp.: + few; ++ moderate number; +++ large number of organisms. The bacteria location 0, absent; 1 = on mucosal surface and within gastric pits; 2 = 1+ lumen of the gastric glands; KI-67: zone 2, isthmus and zone 3, gland base; p53 and CDX2: −, negative.

Of nine GP lesions, seven were classified as hyperplastic polyps (Figure 1B) and two as inflammatory polyps (Figure 1C). No signs of dysplasia were identified. All GPs revealed *Helicobacter* spp. organisms (Figure 1D), preferentially located in the lumen of the gastric glands (Figure 1E) (Table 2).

Differences on COX-2 immunoexpression between NGM and GPs were minimal (Table 2). Both groups presented strong COX-2 expression in more than 75% of gastric superficial epithelium. Deeply, the gastric glands of NGM showed a weak to moderate COX-2 expression, while the glands of GPs showed strong COX-2 expression (Figure 1F). Nevertheless, GPs presented diffuse COX-2 cytoplasm accumulation in epithelial cells.

The proliferative index of GPs was higher (mean value of 24,8%) than NGM (mean value of 20,0%). Ki-67 signal was detected only in the proliferative compartment of NGM whereas in the GPs, this labelling often reached zone 3 (Figure 1G).

Both p53 and CDX2 immunodetection was entirely negative in all cases of canine NGM and GPs (Figure 1H and I).

## Discussion & conclusion

Studies regarding COX-2 in human NGM reported lack [15,16], weak [17] or generalised expression [18]. These discrepancies are perhaps related to the primary antibody (monoclonal or polyclonal) and to the scoring system adopted for immunoreactivity evaluation. The consistent expression of COX-2 in NGM could be explained by two hypotheses: (1) COX-2 is constitutively expressed in canine gastric mucosa; or (2) recent studies showed that *H. pylori* induced COX-2 expression in human gastric mucosa and McCarthy et al. [19] concluded that COX-2 expression in antral mucosa was reduced but not eliminated in the epithelium after successful eradication of *H. pylori*. Sung et al. [15] also showed that though eradication of *H. pylori*, there was only a modest reduction of COX-2 in the gastric epithelium, although COX-2 expression in the lamina propria was markedly reduced. Despite these dogs being currently negative for *Helicobacter* spp., it is not possible to rule out a previous infection by these organisms that could justify the persistent expression of COX-2.

The overexpression of COX-2 in the deeper glands of GPs may be explained by the presence of varying amount of *Helicobacter* organisms in this gastric compartment which, similarly to what happens in humans with *H. pylori,* may lead to an increased expression of this enzyme through the production of proinflammatory cytokines [15-17].

By definition, a GP is a hyperproliferation of the gastric mucosa, thus its onset is necessarily related to an increase in cell turnover and mitotic activity. Yao et al. reported a proliferative index of 22.2% in human hyperplastic GPs. The slightly difference in the percentages obtained in both studies may be related to the species in question (human vs. dog) and/or with sample size (22 vs. 9 cases). Additionally, increased proliferation of gastric mucosa was associated with *H. pylori* [20] since the infection briefly increases apoptosis of surface and proliferative cells and then expands the proliferative zone to deeper in the gland, presumably as a compensatory response [21].

The proliferative zone in the antral stomach is in the lower third of the typical glands units [21], close to the base of the gastric pits, constituting the site of cellular renewal. This very short area, with scant or no mitoses, is almost unidentifiable in inactive gastric mucosa. In human NGM, Ki-67 signal is present only in the proliferative compartment [13] as such in our study (Table 2). However, in the GPs, this labelling often reached zone 3 (Figure 1G), presumably because in the isthmus there are cells that constantly regenerate others that migrate bidirectionally, up to the mucosal surface and down to the gland base, as they differentiate into mature cells [21].

Carrasco et al. [22] described p53-positive staining in approximately 94.1% of canine gastric carcinomas. Our results reinforce previous human studies in which p53 was only recognised in (pre)neoplastic components [9] rather than non-tumoral gastric mucosa [18] or GPs [8]. Furthermore, Guo et al. [2] found co-expression of both CA19-9 and p53 confined to small areas of atypical epithelial cells, estimated as adenocarcinoma, present in a human hyperplastic foveolar polyp. Additionally, the KI-67 index was higher in these malignant foci when compared with the surrounded gastric mucosa.

Doster et al. [23] described CDX2 expression within foci of metaplastic change, namely goblet cells, present in all the cases of canine gastric adenocarcinomas included in their study. IM is an unusual phenomenon in the dog and up to date there is no description concerning its spontaneous occurrence. In humans, IM is usually induced by continuous irritation of the gastric mucosa and *H. pylori* infection remains an important triggering factor. The molecular pathways involved in canine regulation and function of CDX2 are unknown. However, since the expression of the same transcription factor is associated with the maintenance/development of the intestinal phenotype in both humans and dogs, the hypothesis that CDX2 is similarly regulated in both species is plausible (data not published).

*Helicobacter* spp. present in cases of canine GPs may develop different strategies of colonization, infection and pathogenicity from those practised by *H. pylori* in humans, eventually justifying the rarity of IM in the stomach of the dog. Therefore, the pathways responsible for the induction of CDX2 expression in dogs may differ from those already described in humans. Further studies are needed to confirm this hypothesis.

Although certain pathological features of polyps correlate with the risk of developing cancer in humans, in dogs it’s still not possible to determine which lesions will progress to cancer. In conclusion, the present study suggests that canine sporadic GPs are a rare finding and of benign nature.
